# Melatonin Alleviates Glucose and Lipid Metabolism Disorders in Guinea Pigs Caused by Different Artificial Light Rhythms

**DOI:** 10.1155/2020/4927403

**Published:** 2020-10-22

**Authors:** Wei Liu, Yunchao Zhang, Qi Chen, Su Liu, Weilong Xu, Wenbin Shang, Lijuan Wang, Jiangyi Yu

**Affiliations:** ^1^Department of Endocrinology and Metabolism, Jiangsu Province Hospital of Chinese Medicine, Affiliated Hospital of Nanjing University of Chinese Medicine, Nanjing 210029, China; ^2^Key Laboratory for Metabolic Diseases in Chinese Medicine, First College of Clinical Medicine, Nanjing University of Chinese Medicine, Nanjing 210023, China

## Abstract

Modern lifestyle-associated factors, such as high-calorie intake, high-fat diet (HFD), and excessive artificial light, are risk factors for glucose and lipid metabolism disturbances. Melatonin may be beneficial for managing obesity and diabetes; however, the underlying molecular mechanisms are not well elucidated. We aimed to assess whether melatonin has beneficial effects on constant artificial light-induced fat deposition, lipid metabolism, and insulin resistance. Guinea pigs were randomly divided into five experimental groups: control (C), HFD (H), 12 h light (12HL), 24 h light (24HL), and melatonin (M). The majority of indexes, including insulin resistance and obesity, were measured after 10 weeks. AMP-activated protein kinase *α* (AMPK*α*)/peroxisome proliferator-activated receptor-*α* (PPAR*α*) pathway expression was analyzed by quantitative reverse transcription PCR and western blotting. Although insulin resistance and obesity indexes were higher in the 24HL group than in the 12HL group, they were significantly lower in the M group than in the 24HL group. Melatonin treatment markedly upregulated AMPK*α*, phosphorylated AMPK*α* (p-AMPK*α*), PPAR*α*, and carnitine palmitoyl-CoA transferase 1 A (CPT1A) gene and protein expression. Melatonin may alleviate insulin resistance and obesity caused by persistent artificial light exposure in guinea pigs, likely via activation of the AMPK*α*/PPAR*α* signaling pathway.

## 1. Introduction

Type 2 diabetes (T2D) is characterized by persistent hyperglycemia, deficient insulin secretion, or insulin resistance and has become a global public health concern over recent decades [1]. China has a population of 1.38 billion people, with an estimated 110 million patients with diabetes, making it the country with the largest diabetic population [2].

A genome-wide association study showed more than 400 T2D-associated gene variants. These were mostly related to islet function; however, they explained less than 20% of overall disease risk [3, 4]. As we all know, dietary fat is related to excessive energy intake, which may cause T2D and adiposity [5]. Nevertheless, an estimated 20% of the North American and European populations are engaged in some type of nighttime shift work [6, 7]. Other epidemiological studies have shown an increased prevalence of obesity, metabolic syndrome, glucose intolerance, and T2D among individuals engaging in shift work [8, 9]. However, it is unclear whether shift work influences glucose and lipid metabolic balance.

Melatonin (N-acetyl-5-methoxytryptamine, MLT) is a ubiquitous bioactive molecule synthesized in most animals and plants [10], produced primarily by the pineal gland, and influenced by the circadian rhythm and life/dark cycles [11]. Furthermore, melatonin has been used for treating excessive sleepiness, insomnia, or both [12]. It has recently been shown that melatonin may aid in obesity [13, 14] and diabetes management [15, 16]. Most experimental designs focus on how melatonin ameliorates HFD-induced glucose and lipid metabolic imbalance. However, a few studies have examined the effect of different light cycles and circadian rhythms on T2D pathogenesis. Thomas et al. [17] demonstrated that the administration of melatonin increased insulin secretion by the pancreas islets in Sprague-Dawley rats fed an HFD under different light rhythms. However, the effect of melatonin on glucose and lipid metabolism in the liver and adipose tissues remains unclear.

The primary objective of this study was to observe whether melatonin beneficially affects hepatic fat deposition, lipid disorder, and insulin resistance due to the constant exposure to artificial light. Further, we aimed to determine whether these mechanisms are associated with the AMP-activated protein kinase (AMPK) signaling pathway, a cellular energy sensor that reportedly has the potential for regulating metabolic activity [18]. Recent research has indicated that guinea pigs are more suitable models of T2D than conventional mouse or rat rodent models [19]. First, guinea pigs are rodents with abundant cones, accounting for 8–17% of their photoreceptors, and have a sharp vision [20]. Furthermore, owing to the rapid development of their eyeballs, guinea pigs are born with a well-developed visual system [21, 22]. Secondly, guinea pigs share more similarities with human lipid metabolism, including cholesterol metabolism and transport, than any other rodent. Additionally, a greater proportion of their cholesterol is associated with low-density lipoproteins (LDLs) [23, 24]. Therefore, in the current study, guinea pigs were selected as a novel animal model to observe the effects of different light rhythms.

## 2. Materials and Methods

### 2.1. Experimental Animals

Female guinea pigs aged 4 weeks and weighing 150–300 g were purchased from the Laboratory Animal Center of Nanjing University of Chinese Medicine, China. The animals were allowed to acclimatize for at least 6 days before commencing the experiments and were housed under a temperature of 25 ± 3°C and 40–60% humidity. Food and water were available ad libitum. Before initiating experimental protocols, all guinea pigs were synchronized to a standard 12 h light/dark cycle (lights on from 6:00 AM to 6:00 PM). After beginning the experiment, the house light cycle was changed and extended to observe the influence of different light rhythms.

The animal welfare and experimental procedures complied with the guidelines of the Laboratory Animal Center of Nanjing University of Chinese Medicine (Jiangsu, China) and were officially approved by the Ministry of Health, China, in accordance with NIH guidelines (2002). The laboratory procedures were approved by the animal ethics committee of the Nanjing University of Chinese Medicine (animal ethics no. 201905A020).

### 2.2. Reagents

Melatonin was purchased from Sigma Chemical (St. Louis, MO, USA). It was dissolved in ethanol and diluted in 0.9% NaCl saline to obtain a final ethanol concentration of 0.02%.

### 2.3. Experimental Design

Four-week-old female guinea pigs (150–300 g) were randomly divided into five experimental groups (*n* = 12 per group): the C and H groups were exposed to natural light and fed standard chow and high-fat chow, respectively; the 12HL and the 24HL groups were exposed to artificial light (from a halogen lamp, 12 V, 35 W, China) for 12 and 24 hours, respectively, and fed high-fat chow. The M group was the same as the 24HL group but was subcutaneously injected with melatonin (10 mg/kg) daily during weeks 7 to 10. The C, H, and 12HL groups were housed under a 12 h dark/light cycle, whereas the 24HL and M groups received 24 h constant light exposure. All groups received 500 ± 50 lx light intensity.

The standard guinea pig chow provided 70%, 20%, and 10% of calories from carbohydrates, proteins, and fats, respectively. The HFD provided 27.5%, 27.5%, and 45% of calories from carbohydrates, proteins, and fats, respectively. Chow was prepared by the Laboratory Animal Center of Nanjing University of Chinese Medicine, China.

Food intake and water consumption were recorded daily, and body weight was measured weekly. The oral glucose tolerance test (OGTT) (2 g/kg) was performed on days 35 and 65, whereas insulin tolerance test (ITT) (1 U/kg) was performed on days 39 and 70. At the end of the treatment period, animals were fasted for 12 h and anesthetized with 2% pentobarbital sodium (1 mL/kg body weight). The enterocoelia was carefully incised, and approximately 8 mL of blood was drawn from the abdominal aorta followed by decapitation. After whole blood was allowed to rest for 2 h, the serum was collected and centrifuged at 3,000 × g for 15 min at 4°C. Hepatic and perirenal adipose tissue samples were stored in paraformaldehyde at 4°C and refrigerated at -80°C for subsequent analyses.

### 2.4. Food Intake, Water Consumption, and Body Weight Measurement

The guinea pigs were fed daily on a precise feeding schedule (at 8:00 AM) with equal amounts of food and water. Food was provided according to the body weight of each guinea pig, at approximately 6 g of daily chow per 100 g body weight. Approximately 140 mL water was offered to each guinea pig daily. Total food intake and water consumption were measured by subtracting the remaining food and water from the total amount provided. Body weights were monitored weekly in the morning.

### 2.5. Fasting Blood Glucose (FBG) Level Measurement

FBG levels (after 12 h of fasting) were measured every 2 weeks on days 0, 14, 28, 42, 56, and 70. The FBG levels were estimated via commercially available glucose test papers (Jiangsu Yuyue Medical Equipment & Supply Co., Ltd., Nanjing, China) on the basis of the glucose oxidase method, according to the manufacturer's instructions. The results are expressed as millimoles per liter of plasma.

### 2.6. OGTT

All animals were fasted for 12 h followed by intragastric administration of a single dose of 50% glucose at a concentration of 2 g/kg body weight. Blood samples were collected from the middle of the dorsal foot vein of guinea pigs in each group immediately before glucose administration (0 min) and at 30, 60, 90, and 120 min after glucose administration. Plasma glucose levels were determined by commercially available glucose test papers (Jiangsu Yuyue Medical Equipment & Supply Co., Ltd., Nanjing, China). The results are expressed as millimoles per liter of plasma.

### 2.7. ITT

All animals were fasted for 12 h and then intraperitoneally administered with a single dose of insulin at a concentration of 1 IU/kg body weight. Blood samples were collected from each group immediately before (0 min) and at 30, 60, 90, and 120 min after insulin administration. Plasma glucose levels were determined via commercially available glucose test papers (Jiangsu Yuyue Medical Equipment & Supply Co., Ltd., Nanjing, China) and assessed via the glucose oxidase method and are expressed as millimoles per liter of plasma.

### 2.8. Biochemical Analysis

Total glucose (TG), total cholesterol (TC), low-density lipoprotein cholesterol (LDL-C), high-density lipoprotein cholesterol (HDL-C), serum aspartate transferase (AST), and alanine transferase (ALT) levels in the blood serum were determined using an automatic biochemical analyzer (Beckman Coulter, Miami, Florida, USA) in the Department of Laboratory Medicine of Jiangsu Provincial Hospital of Chinese Medicine. The serum levels of fasting insulin (INS), melatonin, adiponectin, leptin, tumor necrosis factor *α* (TNF-*α*), interleukin 6 (IL-6), and IL-1*β* were also measured by enzyme-linked immunosorbent assay (ELISA), according to the manufacturer's instructions. Guinea pig ELISA kits were purchased from Nanjing Jin Yibai Biological Technology Co., Ltd. (Nanjing, China).

Following 4 weeks of melatonin treatment, the HOMA-IR index was calculated using relationships between the blood glucose and insulin levels according to the following equation: HOMA − IR = [fasting serum insulin (mIU/L) × FBG (mmol/L)]/22.5 [25]

### 2.9. Hepatic and Adipose Tissue Pathology

Formalin-fixed and paraffin-embedded tissues, selected from the same parts of the guinea pig livers, were cut into 6 *μ*m thick slices before H&E staining. The optimal cutting temperature-embedded fresh hepatic and adipose tissues were sliced at a thickness of 8 *μ*m. H&E staining was used to determine the overall morphology, whereas Oil Red O staining was used to identify the neutral lipid and fatty acid content. Staining was performed according to the manufacturer's instructions (Wuhan Servicebio Biological Technology Co., Ltd., Wuhan, China). Pathological sections were observed under an optical microscope to reveal the pathological changes. The immunohistochemical staining strength of each group was quantitatively assessed with Image-Pro Plus 5.0 (Media Cybernetics, Silver Spring, MD) software. Quantification of the stained area, namely, the AOD (integrated optical density (IOD)/area in each image), was performed according to a previous study [26].

### 2.10. Quantitative Reverse Transcription Polymerase Chain Reaction (qRT-PCR)

Total RNA was extracted from hepatic and adipose tissues by RNA Trizol reagent (Invitrogen, Carlsbad, CA, USA). The total RNA purity and concentrations were measured three times with an ultraviolet spectrophotometer at an OD260 : 280 ratio of 1.8 : 2.1. Reverse transcription was performed at 50°C for 15 min and 85°C for 5 s to generate cDNA by a HiScript II Q RT SuperMiX for qPCR (Vazyme Biotech Co., Ltd. Nanjing, China). PCR was performed with a ChamQ™ SYBR qPCR Master Mix (Vazyme Biotech Co., Ltd. Nanjing, China) by a 7500 Real-Time PCR system (Applied Biosystems, CA, USA). The thermocycling conditions were set at 1 cycle of predegeneration at 95°C for 30 s, followed by 40 cycles at 95°C for 10 s and 60°C for 30 s. A melting curve was later obtained at 95°C for 15 s, 60°C for 60 s, and 95°C for 15 s. Glyceraldehyde-3-phosphate dehydrogenase (GAPDH) was used as an internal reference. The cycle threshold (Ct) value obtained for each target gene was normalized to that of the GAPDH gene using the formula 2^-*ΔΔ*Ct^. The relative quantity of each gene was expressed as the fold change with respect to the control after normalization to GAPDH. Primer sequences were synthesized by Sangon Biotech (Shanghai) Co., Ltd. The sequences used in this study are shown in Table 1.

### 2.11. Western Blotting Analysis

Fresh hepatic and adipose tissues from guinea pigs were homogenized and centrifuged at 12,000 × g for 5 min at 4°C in RIPA lysis buffer (Beyotime Institute of Biotechnology, Shanghai, China), supplemented with Protease Inhibitor Cocktail (Thermo Scientific, Shanghai, China) and PhosStop Phosphatase Inhibitor Cocktail (Thermo Scientific, Shanghai, China). The total protein was then collected, and protein levels were quantified with a BCA protein assay kit (Beyotime Institute of Biotechnology, Shanghai, China). Proteins (20 mg) were separated by 10% sodium dodecyl sulfate-polyacrylamide gel electrophoresis (Beyotime Institute of Biotechnology, Shanghai, China) and transferred onto polyvinylidene difluoride (PVDF) membranes. The membranes were blocked with 5% skim milk for 1 h and washed three times with Tris-buffered saline Tween-20 (TBST) on a shaking table. Membranes were then incubated overnight at 4°C with specific primary antibodies, including anti-AMPK*α* antibody (diluted 1 : 1000; Sigma, St. Louis, MO, USA), anti-phospho-AMPK-*α*^Thr172^ antibody (diluted 1 : 2000; ABclonal, Wuhan, China), anti-PPAR*α* antibody (diluted 1 : 1000; LifeSpan BioSciences Inc., Seattle WA, USA), anti-CPT1A antibody (diluted 1 : 1000; LifeSpan BioSciences Inc., Seattle WA, USA), and anti-GAPDH antibody (diluted 1 : 5000; Affinity, Cambridgeshire, UK). Next, the membranes were incubated with a horseradish peroxidase-conjugated goat anti-rabbit antibody (diluted 1 : 5000, Affinity, Cambridgeshire, UK) for 1 h at 25°C. After washing three times with TBST, the protein bands were evaluated using a Tanon-5200 Multi Biomolecular Imager (Tanon Science and Technology Co., Ltd., Shanghai, China) and analyzed with Image-Pro Plus 5.0 (Media Cybernetics, Silver Spring, MD). All signals were normalized to those of the housekeeping protein GAPDH.

### 2.12. Statistical Analyses

All statistical analyses were conducted using GraphPad Prism 8.0 software (GraphPad Software, San Diego, CA, USA), and the results are expressed as the mean ± standard deviation. Statistical significance was determined using one-way analysis of variance (ANOVA) with Dunnett's post hoc analysis or two-way ANOVA with Bonferroni's post hoc analysis, according to the number of comparisons and variables. Differences were deemed statistically significant at *P* < 0.05.

## 3. Results

### 3.1. Effect of Melatonin on Food Intake, Water Consumption, Body Weight, FBG and Insulin Levels, and HOMA-IR

**Figure 1:**
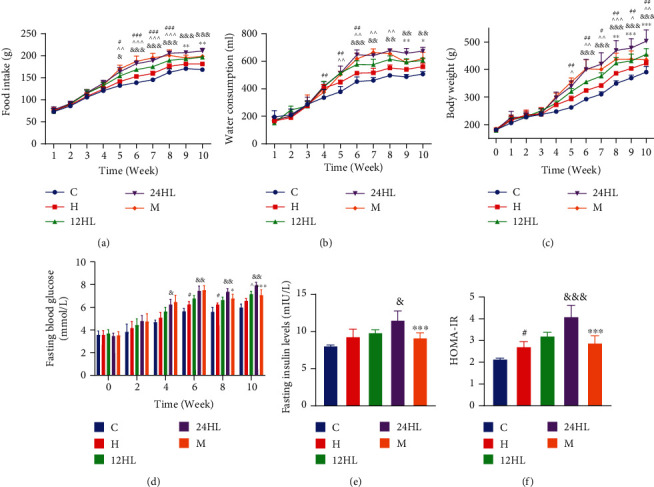
Effect of melatonin on food intake, water consumption, body weight, HOMA-IR, and fasting blood glucose and insulin levels. (a) Food intake, (b) water consumption, (c) body weight, (d) fasting blood glucose, (e) fasting insulin level, and (f) HOMA-IR. C: control group; H: HFD group; 12HL: 12 h light group; 24HL: 24 h light group; M: melatonin group. The values are expressed as the means ± S.D. (*n* = 7–12 per group). ^#^*P* < 0.05, ^##^*P* < 0.01, and ^###^*P* < 0.001, HFD vs. control; ^^^*P* < 0.05, ^^^^*P* < 0.01, and ^^^^^*P* < 0.001, 12HL vs. HFD group; ^&^*P* < 0.05, ^&&^*P* < 0.01, and ^&&&^*P* < 0.001, 24HL vs. 12HL group; ^∗^*P* < 0.05, ^∗∗^*P* < 0.01, and ^∗∗∗^*P* < 0.001, M vs. 24HL group.

During the first 3 weeks of 10-week monitoring, the control (C), high-fat diet (H), 12 h light (12HL), 24 h light (24HL), and melatonin (M) groups did not show significantly different food intake, water consumption, body weight, or FBG levels. However, statistically significant differences in these parameters gradually began to emerge at weeks 4 and 5 for the C, H, 12HL, and 24HL groups (*P* < 0.05 and *P* < 0.01; Figures 1(a)*–*1(d)). Furthermore, food intake, water consumption, and body weight significantly differed between the C and H groups (*P* < 0.001, *P* < 0.01, and *P* < 0.01, respectively) and between the H and 12HL groups (*P* < 0.001, *P* < 0.01, and *P* < 0.01, respectively), as well as between the 12HL and 24HL groups (*P* < 0.001, *P* < 0.001, and *P* < 0.001, respectively) in week 6. FBG levels in the H group were significantly greater than those in the C group (*P* < 0.05), as well as in the 24HL group compared to the 12HL group (*P* < 0.01). However, food intake, water consumption, body weight, and FBG levels in the M group were not significantly different from those in the 24HL group before melatonin administration. After melatonin administration (10 mg/kg) to the M group, from week 7 onward, food intake and water consumption significantly decreased from weeks 9 to 10 relative to those of the 24HL group (*P* < 0.01). The body weight and FBG levels of the M group gradually significantly decreased from weeks 8 to 10 (*P* < 0.01 and *P* < 0.001, respectively) (Figures 1(a)–1(d)).

After 4 weeks of treatment, the fasting insulin levels of guinea pigs were measured, and the homeostatic model assessment of insulin resistance (HOMA-IR) score was estimated. Although the fasting insulin levels in the H group were not different from those in the C group (*P* > 0.05), the H group had a significantly greater HOMA-IR score (*P* < 0.05; Figures 1(e) and 1(f)). In addition, the fasting insulin level and HOMA-IR score did not significantly differ between the H and 12HL groups (*P* > 0.05). However, the fasting insulin level and HOMA-IR score in the 24HL group were greater than those of the 12HL group (*P* < 0.05 and *P* < 0.001, respectively). These were also significantly lower in the M group than in the 24HL group (*P* < 0.001; Figures 1(e) and 1(f)).

### 3.2. Effect of Melatonin on OGTT and ITT before or after Treatment

**Figure 2:**
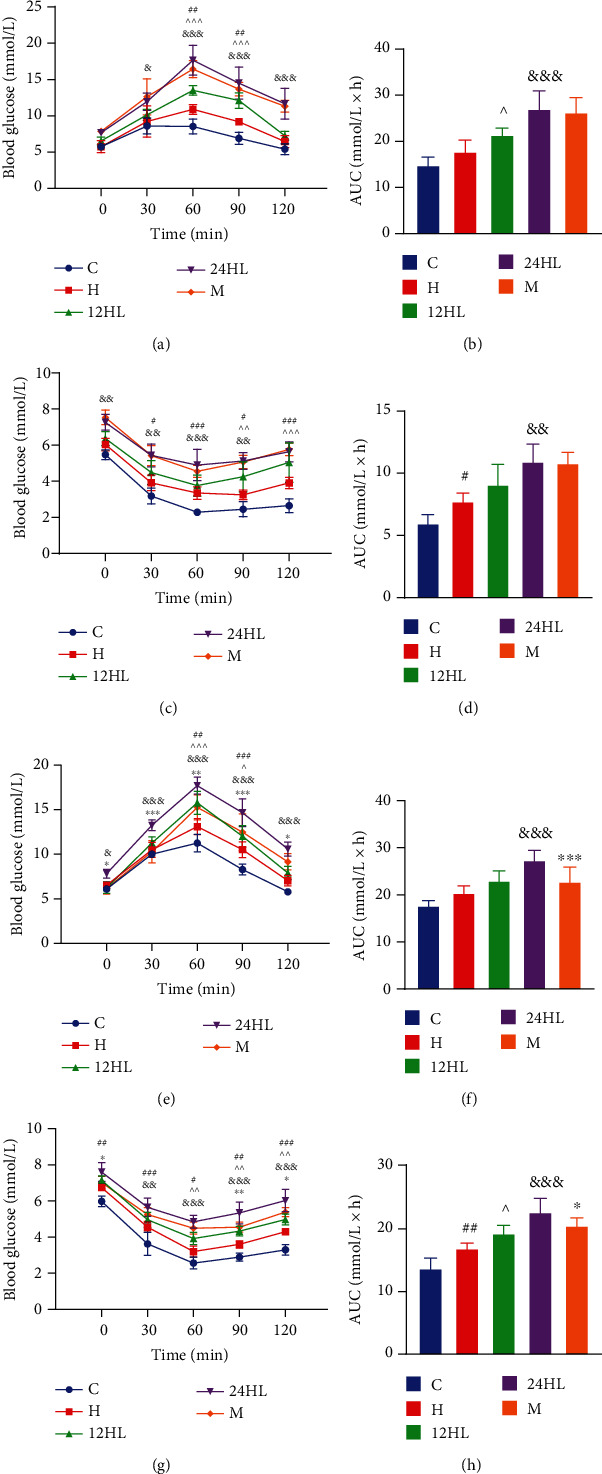
Effect of melatonin on OGTT and ITT before or after treatment. (a) OGTT, (b) AUC of OGTT, (c) ITT, and (d) AUC of ITT before treatment (on week 6); (e) OGTT, (f) AUC of OGTT, (g) ITT, and (h) AUC of ITT after treatment (on week 10). C: control group; H: HFD group; 12HL: 12 h light group; 24HL: 24 h light group; M: melatonin group. The values are expressed as the means ± S.D. (*n* = 7-12 per group). ^#^*P* < 0.05, ^##^*P* < 0.01, and ^###^*P* < 0.001, HFD vs. control; ^^^*P* < 0.05, ^^^^*P* < 0.01, and ^^^^^*P* < 0.001, 12HL vs. HFD group; ^&^*P* < 0.05, ^&&^*P* < 0.01, and ^&&&^*P* < 0.001, 24HL vs. 12HL group; ^∗^*P* < 0.05, ^∗∗^*P* < 0.01, and ^∗∗∗^*P* < 0.001, M vs. 24HL group.

To assess the effect of melatonin on systemic glucose homeostasis, we performed OGTT and ITT. OGTT showed significant and persistent glucose intolerance in the 24HL group (*P* < 0.05 for the 24HL vs. 12HL group at 30 min; *P* < 0.001 for the 24HL vs. 12HL group at 60, 90, and 120 min after glucose administration; Figure 2(a)). Further, the ITT results revealed markedly reduced insulin sensitivity in the 24HL group at 6 weeks (*P* < 0.01 for the 24HL vs. 12HL group at 0, 30, and 90 min; *P* < 0.001 for the 24HL vs. 12HL group at 60 min following insulin administration; Figure 2(c)). Additionally, the areas under the curve (AUC) for blood glucose during the OGTT and ITT of the 12HL groups were substantially lower than those of the 24HL group (*P* < 0.001 and *P* < 0.01, respectively; Figures 2(b) and 2(d)). The M group did not significantly differ from the 24HL group before melatonin treatment.

After 4 weeks of treatment, the OGTT and ITT were again performed to estimate the degree of insulin resistance. The OGTT of the M group (administered melatonin) had significantly less insulin resistance than that of the 24HL group (*P* < 0.05 for the M vs. 24HL group at 0 and 120 min; *P* < 0.001 for the M vs. 24HL group at 30 and 90 min; and *P* < 0.01 for the M vs. 24HL group at 60 min postglucose administration; Figure 2(e)). Additionally, the ITT of the M group exhibited relatively stronger responses to insulin than that of the 24HL group (*P* < 0.05 for the M vs. 24HL group at 0 and 120 min; *P* < 0.01 for the M vs. 24HL group at 90 min postinsulin administration; Figure 2(g)). Lastly, the AUC for blood glucose during the M group OGTT was significantly lower than that of the 24HL group (*P* < 0.001; Figure 2(f)), as was the AUC for blood glucose during the M group ITT (*P* < 0.05; Figure 2(h)).

### 3.3. Effect of Melatonin on Blood Lipid Metabolism

**Figure 3:**
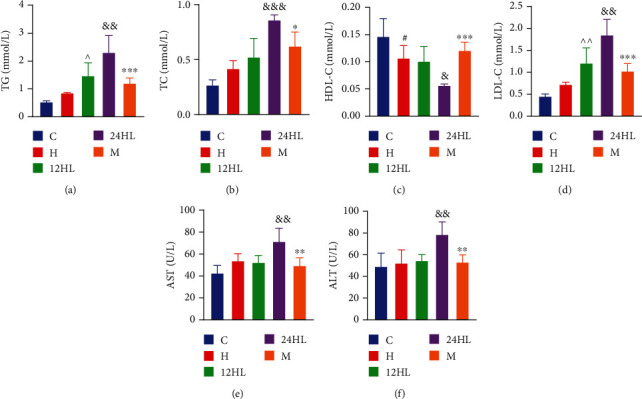
Effect of melatonin on blood lipid metabolism. (a) TG, (b) TC, (c) HDL-C, (d) LDL-C, (e) AST, and (f) ALT after treatment (on week 10). C: control group; H: HFD group; 12HL: 12 h light group; 24HL: 24 h light group; M: melatonin group. The values are expressed as the means ± S.D. (*n* = 6-8 per group). ^#^*P* < 0.05, HFD vs. control; ^^^*P* < 0.05, and ^^^^*P* < 0.01, 12HL vs. HFD group; ^&^*P* < 0.05, ^&&^*P* < 0.01, and ^&&&^*P* < 0.001, 24HL vs. 12HL group; ^∗^*P* < 0.05, ^∗∗^*P* < 0.01, and ^∗∗∗^*P* < 0.001, M vs. 24HL group.

To determine whether melatonin affects the blood lipid content of guinea pigs under different light rhythms, we assessed the levels of different types of lipids. The TG, TC, and LDL-C levels were significantly higher in the 24HL group than in the 12HL group (*P* < 0.01, *P* < 0.001, and *P* < 0.01, respectively), whereas levels in the M group were the lowest (*P* < 0.001, *P* < 0.05, and *P* < 0.001, respectively, for the M group vs. 24HL group, after melatonin treatment; Figures 3(a), 3(b), and 3(d)). HDL-C levels were lower in the H group than in the C group (*P* < 0.05; Figure 3(c)) and were also lower in the 24HL group than in the 12HL group (*P* < 0.05; Figure 3(c)). After melatonin treatment, HDL-C levels were much greater in the M group than in the 24HL group (*P* < 0.001; Figure 3(c)). Lipid metabolism therefore appeared to become disordered with increasing duration of light exposure. However, melatonin effectively recovered the lipid balance.

Serum AST and ALT levels were determined to evaluate liver function. AST and ALT levels were higher in the 24HL group than in the 12HL group (*P* < 0.01). The elevated levels of AST and ALT demonstrated that long-term lipid metabolism disturbance could result in damage to hepatocytes. However, AST and ALT levels were lower in the M group after melatonin treatment (*P* < 0.01; Figures 3(e) and 3(f)).

### 3.4. Effect of Melatonin on Endocrine Hormones and Inflammation

**Figure 4:**
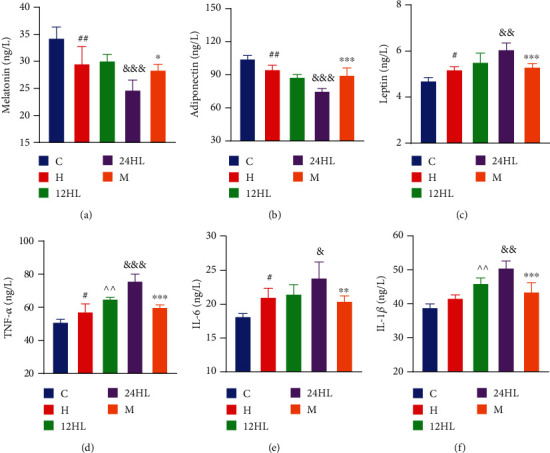
Effect of melatonin on endocrine hormones and inflammation. (a) Melatonin, (b) adiponectin, (c) leptin, (d) TNF-*α*, (e) IL-6, and (f) IL-1*β* after treatment (on week 10). C: control group; H: HFD group; 12HL: 12 h light group; 24HL: 24 h light group; M: melatonin group. The values are expressed as the means ± S.D. (*n* = 6-8 per group). ^#^*P* < 0.05 and ^##^*P* < 0.01, HFD vs. control; ^^^^*P* < 0.01, 12HL vs. HFD group; ^&^*P* < 0.05, ^&&^*P* < 0.01, and ^&&&^*P* < 0.001, 24HL vs. 12HL group; ^∗^*P* < 0.05, ^∗∗^*P* < 0.01, and ^∗∗∗^*P* < 0.001, M vs. 24HL group.

The level of serum melatonin was significantly lower in the H group (*P* < 0.01) than in the C group. Serum melatonin in the 24HL group was lower than that in the 12HL group (*P* < 0.001), but serum melatonin was significantly higher in the M group than in the 24HL group (*P* < 0.05; Figure 4(a)). Adiponectin and leptin levels were determined to evaluate lipid accumulation. The level of adiponectin in the H group was significantly lower (P <0.01), and the 24HL group showed lower levels of adiponectin than those in the 12HL group (*P* < 0.001). However, the M group exhibited significantly higher adiponectin levels than the 24HL group (*P* < 0.001; Figure 4(b)). Leptin levels in the H group were higher than in the C group (*P* < 0.05), and the 24HL group also had higher leptin levels than the 12HL group (*P* < 0.01). The M group had significantly lower leptin levels than those in the 24HL group (*P* < 0.001; Figure 4(c)). The 24HL group, therefore, had severe leptin resistance, whereas melatonin could alleviate leptin accumulation and elevate adiponectin levels.

Tumor necrosis factor *α* (TNF-*α*), interleukin 6 (IL-6), and IL-1*β* are biomarkers of inflammation. As presented in Figures 4(d)–4(f), TNF-*α*, IL-6, and IL-1*β* levels were higher in the 24HL group than in the 12HL group (*P* < 0.001, *P* < 0.05, and *P* < 0.01, respectively), whereas these decreased significantly in the M group (*P* < 0.001, *P* < 0.01, and *P* < 0.001, respectively). The results show that, in guinea pigs, melatonin could reduce inflammatory cytokine levels due to prolonged light exposure.

### 3.5. Effects of Melatonin on Histopathological Changes in Hepatic and Adipose Tissue

**Figure 5:**
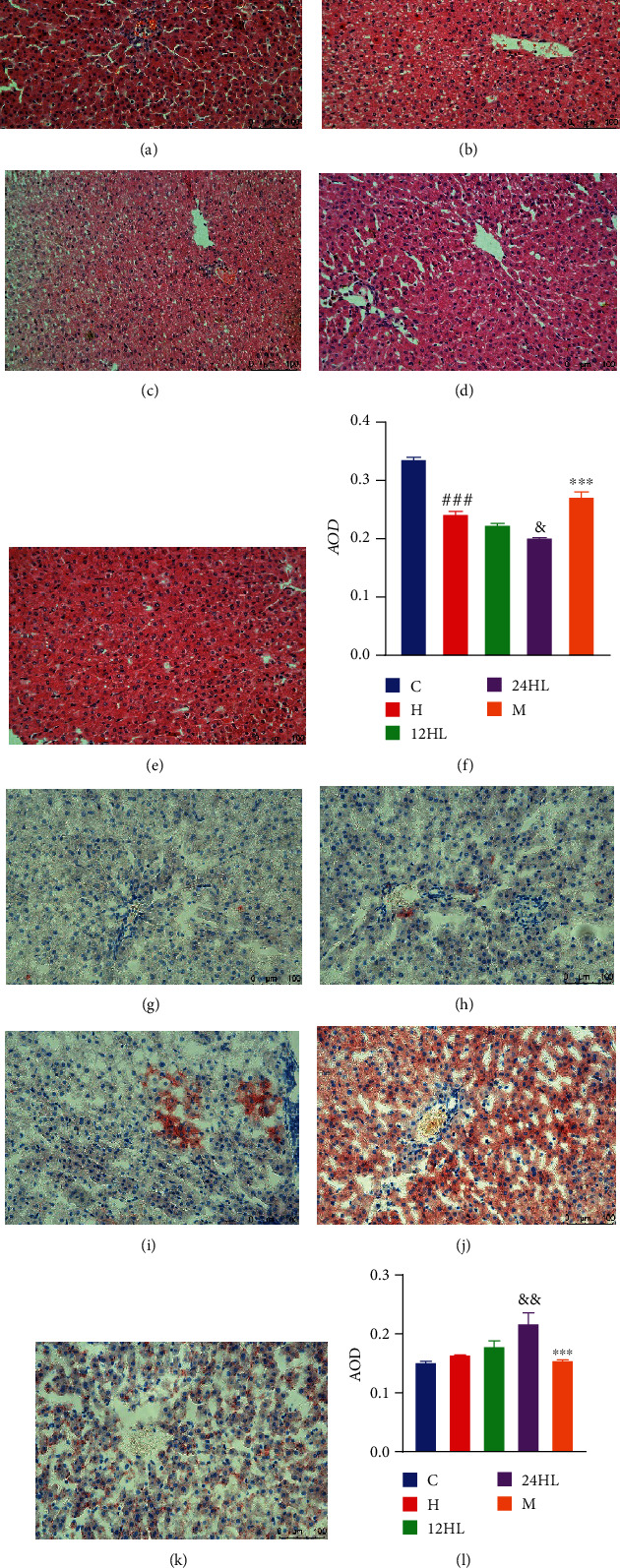
Effects of melatonin on histopathological changes in hepatic tissue. H&E staining (200x): (a) C group, (b) H group, (c) 12HL group, (d) 24HL group, and (e) M group after treatment (on week 10); (f) AOD of H&E staining. Oil Red O staining (200x): (g) C group, (h) H group, (i) 12 HL group, (j) 24HL group, and (k) M group after treatment (on week 10); (l) AOD of Oil Red O staining. Integrated optical density (IOD)/area of staining in each immunohistochemistry staining image. AOD: average optical density; C: control group; H: HFD group; 12HL: 12 h light group; 24HL: 24 h light group; M: melatonin group. The values are expressed as the means ± S.D. (*n* = 3 per group). ^###^*P* < 0.001, HFD vs. control; ^&^*P* < 0.05 and ^&&^*P* < 0.01, 24HL vs. 12HL group; ^∗∗∗^*P* < 0.001, M vs. 24HL group.

Hematoxylin and eosin (H&E) staining of liver sections is shown in Figures 5(a)–5(e); and quantification of the average optical density (AOD) in each image was conducted for further analysis. The hepatocytes of C group guinea pigs showed clear structures with no evidence of steatosis (adipose hollow space) or inflammation, while the H group showed varying degrees of both pathological conditions. In contrast, the 24HL group had more adipose hollow spaces than the 12HL group, while the M group showed less adipose hollow spaces than the 24HL group. Further, steatosis and inflammation increased in the liver, leading to liver cell atrophy and death. Thus, the AOD of the H group was lower than that of the C group (*P* < 0.001), and the AOD of the 24HL group was lower than that of the 12HL group (*P* < 0.05). However, the AOD of the M group was higher than that of the 24HL group (*P* < 0.001; Figure 5(f)). Oil Red O staining of liver tissues is shown in Figures 5(g)–5(k). The C and H groups showed few red lipid droplets, while the 24HL group had swollen and suffused red droplets of varying sizes. Lipid droplets in the M group were markedly less abundant than those in the 24HL group. Oil Red O staining paralleled lipid droplet abundance, and the AOD of the 24HL group was higher than that of the 12HL group (*P* < 0.01). However, the AOD of the M group was lower than that of the 24HL group (*P* < 0.001; Figure 5(l)).

**Figure 6:**
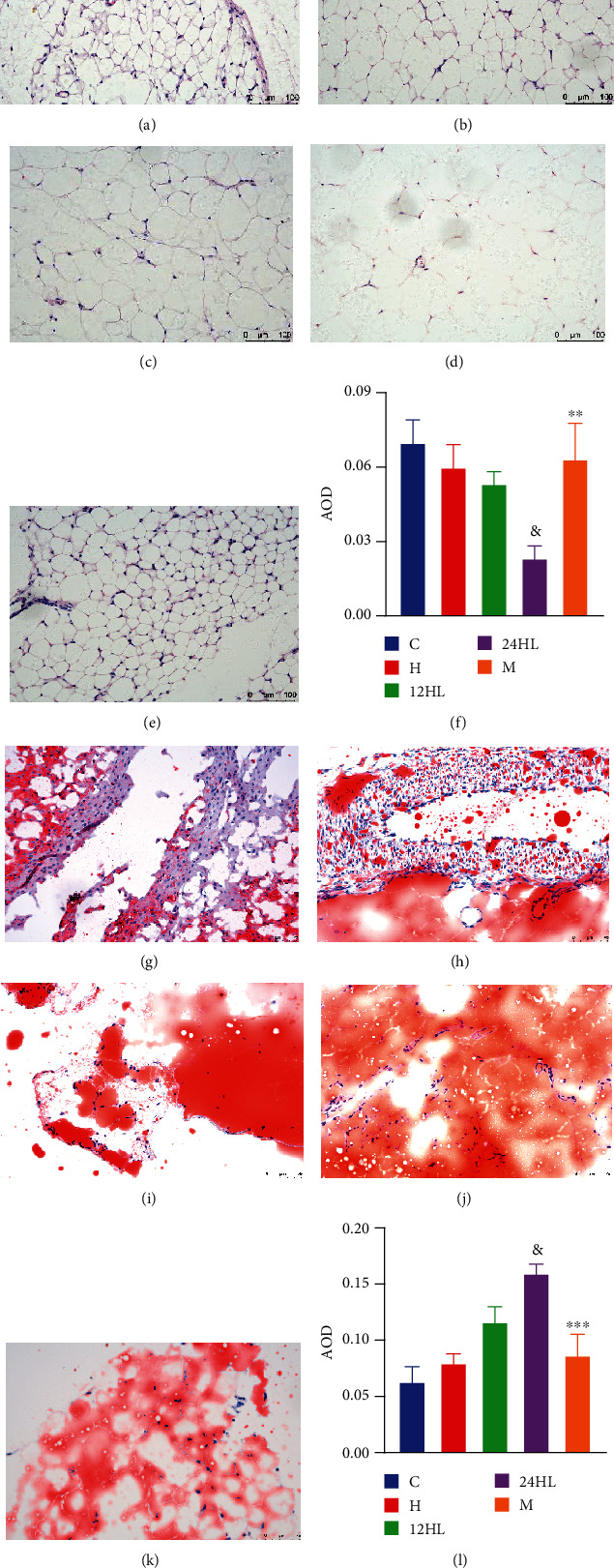
Effects of melatonin on histopathological changes in adipose tissue. H&E staining (200x): (a) C group, (b) H group, (c) 12HL group, (d) 24HL group, and (e) M group after treatment (on week 10); (f) AOD of H&E staining. Oil Red O staining (200x): (g) C group, (h) H group, (i) 12 HL group, (j) 24HL group, and (k) M group after treatment (on week 10); (l) AOD of Oil Red O staining. Integrated optical density (IOD)/area of staining in each immunohistochemistry staining image. AOD: average optical density; C: control group; H: HFD group; 12HL: 12 h light group; 24HL: 24 h light group; M: melatonin group. The values are expressed as the means ± S.D. (*n* = 3 per group). ^&^*P* < 0.05, 24HL vs. 12HL group; ^∗∗^*P* < 0.01 and ^∗∗∗^*P* < 0.001, M vs. 24HL group.

H&E staining and Oil Red O staining were performed on the adipose tissue from each group. Consistent with the changes observed in hepatic tissue, melatonin treatment (M group) decreased the sizes of perirenal adipocytes (Figure 6(e)), and red lipid droplets within these perirenal adipocytes were reduced (Figure 6(k)). Lipid accumulation in adipose cells leads to the formation of larger adipocytes with unclear borders. The AOD of adipose H&E staining of the 24HL group was lower than that of the 12HL group (*P* < 0.05); however, the AOD of the M group exceeded that of the 24HL group (*P* < 0.01; Figure 6(f)). The AOD of adipose Oil Red O staining in the 24HL group was higher than that of the 12HL group (*P* < 0.05), while the AOD of the M group was significantly reduced, as shown in Figure 6(l) (*P* < 0.001).

### 3.6. Effects of Melatonin on AMPK Signaling Pathway-Related mRNA and Protein Expression Levels in Guinea Pig Hepatic Tissue

**Figure 7:**
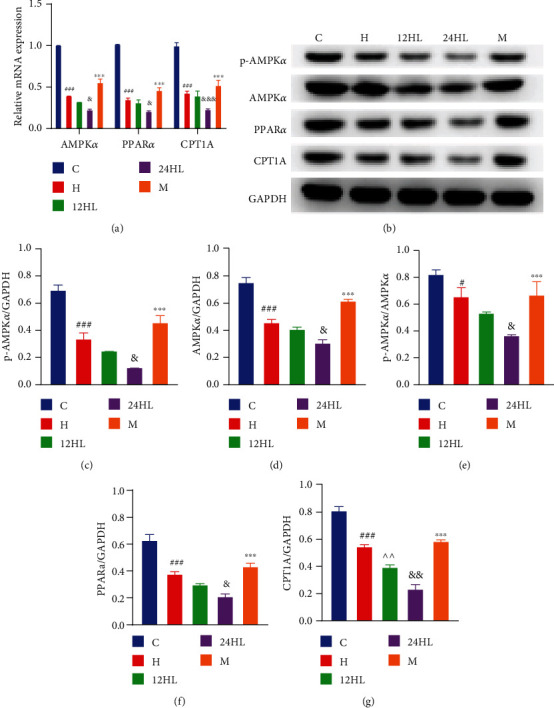
Effects of melatonin on AMPK signaling pathway-related mRNA and protein expression levels in guinea pig hepatic tissue after treatment, at week 10. (a) AMPK*α*, PPAR*α*, and CPT1A mRNA expression as revealed by quantitative reverse-transcription PCR. (b) Western blotting of p-AMPK*α*, AMPK*α*, PPAR*α*, and CPT1A. p-AMPK*α* (c), AMPK*α* (d), p-AMPK*α*/AMPK*α* (e), PPAR*α* (f), and CPT1A (g) protein expression was quantitated by densitometry analysis of the bands. Values were normalized against that for GAPDH and analyzed with ImageJ software. C: control group; H: HFD group; 12HL: 12 h light group; 24HL: 24 h light group; M: melatonin group; AMPK*α*: AMP-activated protein kinase *α*; p-AMPK*α*: phosphorylated AMPK*α*; p-AMPK/AMPK: protein ratio between p-AMPK and total-AMPK; PPAR*α*: peroxisome proliferator-activated receptor-*α*; CPT1A: carnitine palmitoyl-CoA transferase 1 A; GAPDH: glyceraldehyde-3-phosphate dehydrogenase. The values are expressed as the means ± S.D. (*n* = 3 per group). ^#^*P* < 0.05 and ^###^*P* < 0.001, HFD vs. control; ^^^^*P* < 0.01, 12HL vs. HFD group; ^&^*P* < 0.05, ^&&^*P* < 0.01, and ^&&&^*P* < 0.001, 24HL vs. 12HL group; ^∗∗∗^*P* < 0.001, M vs. 24HL group.

AMPK*α*, PPAR*α*, and CPT1A mRNA expression levels were significantly lower in the H group than in the C group (*P* < 0.001; Figure 7(a)). Displaying the effects of different light rhythms, the AMPK*α* and PPAR*α* mRNA expression levels were lower in the 24HL group than in the 12HL group (*P* < 0.05). The mRNA of CPT1A was lower in the 24HL group than in the 12HL group (*P* < 0.001), as shown in Figure 7(a). The AMPK*α*, PPAR*α*, and CPT1A mRNA expression levels were significantly higher after melatonin treatment in the M group (*P* < 0.001; Figure 7(a)) than in the 24HL group.

Consistent with the changes in mRNA levels observed in hepatic tissue, the p-AMPK*α*, AMPK*α*, PPAR*α*, and CPT1A protein expression levels were significantly lower in the H group than in the C group (*P* < 0.001), the protein ratio between p-AMPK and total-AMPK (p-AMPK/AMPK) was also decreased (*P* < 0.05; Figure 7(b)–7(g)). The p-AMPK*α*, AMPK*α*, and PPAR*α* protein expression levels and p-AMPK/AMPK were lower in the 24HL group than in the 12HL group (*P* < 0.05), and the CPT1A protein expression was significantly lower in the 24HL group than in the 12HL group (*P* < 0.01; Figures 7(b)–7(g)). The p-AMPK*α*, AMPK*α*, PPAR*α*, and CPT1A protein expression and p-AMPK/AMPK were significantly increased by melatonin treatment in the M group (*P* < 0.001; Figures 7(b)–7(g)). AMPK signaling pathway-related mRNA and protein expression levels indicated that this pathway might be inhibited in the liver of guinea pigs and that melatonin could be responsible for its activation.

### 3.7. Effects of Melatonin on AMPK Signaling Pathway-Related mRNA and Protein Expression Levels in Guinea Pig Adipose Tissue

**Figure 8:**
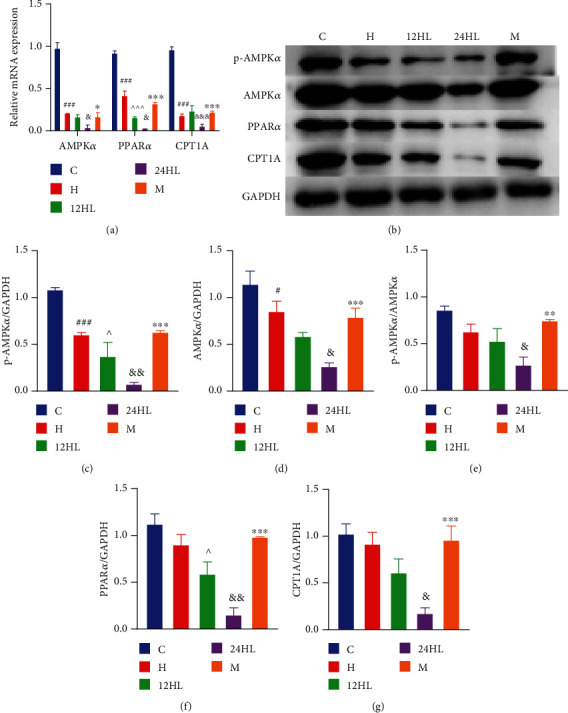
Effects of melatonin on AMPK signaling pathway-related mRNA and protein expression levels in guinea pig adipose tissue after treatment, at week 10. (a) AMPK*α*, PPAR*α*, and CPT1A mRNA expression as revealed by quantitative reverse-transcription PCR. (b) Western blotting of p-AMPK*α*, AMPK*α*, PPAR*α*, and CPT1A. p-AMPK*α* (c), AMPK*α* (d), p-AMPK*α*/AMPK*α* (e), PPAR*α* (f), and CPT1A (g) protein expression was quantitated by densitometry analysis of the bands. Values were normalized against that for GAPDH and analyzed with ImageJ software. C: control group; H: HFD group; 12HL: 12 h light group; 24HL: 24 h light group; M: melatonin group; AMPK*α*: AMP-activated protein kinase *α*; p-AMPK*α*: phosphorylated AMPK*α*; p-AMPK/AMPK: protein ratio between p-AMPK and total-AMPK; PPAR*α*: peroxisome proliferator-activated receptor-*α*; CPT1A: carnitine palmitoyl-CoA transferase 1 A; GAPDH: glyceraldehyde-3-phosphate dehydrogenase. The values are expressed as the means ± S.D. (*n* = 3 per group). ^#^*P* < 0.05 and ^###^*P* < 0.001, HFD vs. control; ^^^*P* < 0.05 and ^^^^^*P* < 0.001, 12HL vs. HFD group; ^&^*P* < 0.05, ^&&^*P* < 0.01, and ^&&&^*P* < 0.001, 24HL vs. 12HL group; ^∗^*P* < 0.05 and ^∗∗∗^*P* < 0.001, M vs. 24HL group.

The AMPK*α*, PPAR*α*, and CPT1A mRNA expression levels were significantly lower in the H group than in the C group (*P* < 0.001; Figure 8(a)). Displaying the effects of different light rhythms, the AMPK*α* and PPAR*α* mRNA expression levels were lower in the 24HL group than in the 12HL group (*P* < 0.05; Figure 8(a)). CPT1A mRNA levels were also significantly lower in the 24HL group (*P* < 0.001), as shown in Figure 8(a). In the M group, AMPK*α* mRNA expression levels were enhanced by melatonin treatment (*P* < 0.05) compared to the 24HL group. PPAR*α* and CPT1A mRNA levels were also significantly boosted by melatonin (*P* < 0.001; Figure 8(a)).

Under a similar 12 h dark/light cycle, when different diets were provided, p-AMPK*α* protein expression was significantly lower in the H group than in the C group (*P* < 0.001). A similar difference was observed for AMPK*α* protein expression (*P* < 0.05). Although the PPAR*α* and CPT1A protein expression levels and p-AMPK/AMPK did not significantly differ between the H and C groups, they exhibited a decreasing trend in the H group. The p-AMPK*α* and PPAR*α* protein expression levels were significantly lower in the 24HL group than in the 12HL group (*P* < 0.01), as was AMPK*α*, CPT1A protein expression, and p-AMPK/AMPK (*P* < 0.05) (Figures 8(b)–8(g)). However, p-AMPK*α*, AMPK*α*, PPAR*α*, and CPT1A protein expression was significantly higher in the M group than in the 24HL group (*P* < 0.001). Furthermore, p-AMPK/AMPK was also increased in the M group relative to the 24H group (*P* < 0.01).

Notably, a 24 h constant light exposure can cause severe imbalances in glucose and lipid metabolism, and AMPK signaling pathway activation was inhibited in the 24HL group. Inhibition of the AMPK signaling pathway was alleviated after melatonin treatment, demonstrating that melatonin may alleviate glucose and lipid disorder.

## 4. Discussion

T2D has become a widely prevalent disease that poses severe threats to human health worldwide [27]. Sleep deprivation, shift work, or exposure to excessive artificial light has become inherent features of a modern lifestyle and is correlated with an increased incidence of T2D and metabolic syndrome [28, 29]. In this study, we simulated changes in the light environment to observe glucose and lipid metabolism in guinea pigs. Using our experimental methods, we preliminarily revealed that melatonin ameliorates glucose and lipid metabolism balance.

The results showed that artificial 24 h continuous light exposure could aggravate the degree of insulin resistance and lipid disorders, compared with an artificial 12 h dark/light cycle. However, insulin resistance and lipid disorders were improved after melatonin administration, and the underlying reasons could be related to two main aspects. On the one hand, melatonin can decrease food intake by improving leptin resistance. The level of melatonin was reduced under continuous light, which could decrease leptin sensitivity, leading to leptin resistance [30]. Leptin is mainly secreted by white adipose tissue and regulates energy homeostasis by inhibiting food intake [31]. Our results showed that the leptin level in the 24HL group exceeded that of the 12HL group, implying that leptin resistance could be generated in response to persistent artificial light exposure (Figure 4). Besides, the leptin level in the M group was lower than that in the 24HL group after 4 weeks of melatonin administration (Figure 4). Thus, melatonin administration could increase leptin sensitivity and restrict food intake. Sinkalu et al. [32] also demonstrated that melatonin administration to broiler chickens decreased feed consumption. On the other hand, melatonin administration could improve the degree of insulin resistance, which could be related to increased fatty acid oxidation via AMPK*α*/PPAR*α* signaling pathway activation.

AMPK is a ubiquitously expressed serine/threonine-protein kinase and is an essential molecular protein for maintaining the balance of energy metabolism. AMPK is mainly composed of the three molecular subunits *α*, *β*, and *γ* [33] and generally exists in the form of a trimer. The *α* subunit mainly plays a catalytic role, whereas the *β* and *γ* subunits maintain trimer stability and ensure substrate specificity [34]. The N-terminal subunit contains a conserved Ser/Thr kinase region and threonine (Thr-172) region, which is an important site for AMPK*α* phosphorylation [35]. Moreover, the liver is one of the central organs most closely associated with energy metabolism. Hepatic AMPK*α* is vital for preventing lipid accumulation in the liver and insulin resistance, maintaining mitochondrial function in brown adipose tissue, and protecting against hypothermia, obesity, and insulin resistance [36].

PPAR*α* is a nuclear receptor that positively regulates fatty acid utilization and catabolism [37]. Generally, AMPK*α* can regulate the expression of PPAR*α*. After its activation by AMPK*α*, PPAR*α* reduces the levels of fatty acids required for triglyceride synthesis by enhancing the expression of CPT1A, a rate-limiting enzyme in fatty acid *β*-oxidation in the mitochondria [38, 39]. Our results showed that AMPK*α*, p-AMPK*α*, PPAR*α*, and CPT1A protein and gene expression were downregulated by increasing artificial light exposure (24HL group) in both hepatic and adipose tissues. However, AMPK*α*, p-AMPK*α*, PPAR*α*, and CPT1A protein and gene expression were upregulated after melatonin treatment (M group). Therefore, melatonin alleviates glycolipid metabolism disorders by activating the AMPK*α*/PPAR*α* signaling pathway, reducing lipid deposition in the liver and adipose tissues, and increasing insulin sensitivity. However, the study had certain limitations. Firstly, many parameters, such as FBG, insulin, OGTT, blood lipids, and Oil Red O staining in the liver and adipose tissues, were not significantly different between the H and C groups; however, they were slightly higher in the H group. Since the diet of guinea pigs mostly includes crude fiber, considering model heterogeneity, it is possible that a few animals in the H group may have not consumed enough HFD, which could, in turn, have influenced the statistical results. Secondly, the muscle is another vital tissue involved in glucose and lipid metabolism that was not examined in the current study; this will be examined in future research. Thirdly, owing to the limited availability of guinea pig-specific antibodies, other proteins associated with insulin resistance, including phosphatidylinositol 3-kinase (PI3K), were not detected. Future studies will, therefore, seek to quantify relevant genes and proteins via proteomic techniques more comprehensively.

## 5. Conclusions

In the view of above discussion, our results demonstrated that, in guinea pigs, persistent artificial light exposure increased the rate of insulin resistance and obesity by downregulating the AMPK*α*/PPAR*α* signaling pathway. Furthermore, melatonin may alleviate insulin resistance and obesity by activating the AMPK*α*/PPAR*α* signaling pathway.

## Figures and Tables

**Table 1:** Gene-specific primers used in this study.

Gene name	Forward primer (5′⟶3′)	Reverse primer (5′⟶3′)
GAPDH	ATCTGACCTGCCGCCTGGAG	AACCTGGTCCTCGGTGTAGCC
AMPK*α*	GTGGTAGCTGTGGTTGCATTGTTC	AGTTGTGAAGGACCGCATGAGTTG
PPAR*α*	CAACCTCACGGAGTTCGCCAAG	GTGGAGGACAGCATGGTGAAGATG
CPT1A	TTGAGGAACACGGCAAGATGAGC	GCGGCTTCACTGATTCCAGATACC

GAPDH: glyceraldehyde-3-phosphate dehydrogenase; AMPK*α*: AMP-activated protein kinase *α*; PPAR*α*: peroxisome proliferator-activated receptor-*α*; CPT1A: carnitine palmitoyl-CoA transferase 1 A.

## Data Availability

The datasets used to support the findings of this study are available from the corresponding authors upon request.
